# Supplementary material for: Serum levels of NLRC4 and MCP-2/CCL8 in patients with active Crohn’s disease
Source: PLoS One. 2021 Nov 17;16(11):e0260034. doi: 10.1371/journal.pone.0260034 (PMC8598053; doi:10.1371/journal.pone.0260034)
Supplement: Attachment — Submitted filename: Response.docx [file pone.0260034.s002.docx]

**Reviewer #1**: The authors studied – for the first time - the serum levels of MCP2/CCL8 and NLRC4 in active CD and found significant differences compared with the control group. This finding could be useful in clinical practice, both in diagnostic and therapy of CD.

I have some suggestions for the authors:

1. The Montreal classification of CD (location, behavior) – has no relevance in this study.

The data of the study is presented in an additional excel file.

2. Please be more precious regarding the selection of the control group; as there are significant differences regarding age and sex it would be useful to check if there are any variation of studied inflammatory markers in general population related to age and sex.

As far as we examined, inflammatory marker variation between different gender and age groups has not been clearly demonstrated. Even so, the difference in age and gender frequency between the case group and the control group in our study may have affected the results. We mentioned this situation in the limitations section.

3. There are some already known inflammatory markers (CRP, RDW, WBC, neutrophils, NLR, PLR, platelets) found to be different from control group but this is not relevant in this study; it would be interesting to compare these known markers with MCP2/CCL8 and NLRC4.

Correlation analyses were added as suggested

4. It is well known that it is no correlation between CDAI and mucosal inflammation; maybe adding calprotectin evaluation will add additional value of the study.

Very few of the patients included in the study have calprotectin data, which is not sufficient for analysis.

5. Some items in the discussion section are not supported by the results (“This supports the role of neutrophils in regulating leukocyte activation and migration in the intestinal damage, inflammation in general, and also as an indicator of disease severity”).

The relevant part has been removed

6. The treatment may play a role in levels of MCP2/CCL8 and NLRC4. Although the patients had active CD only 31 had treatment. Please explain.

Although 31 patients received treatment, they applied with disease activation. Other patients were diagnosed with CD at the time of enrollment in the study and have not received any treatment yet. The possible effect of medication differences between the groups on the results is added to the limitations section.
